# Treatment of Empyema by Antiseptic Injections and Drainage Tubes

**Published:** 1876-10

**Authors:** W. H. Vittum

**Affiliations:** Baraboo, Wis.


### ON THE TREATMENT OF EMPYEMA BY ANTISEPTIC INJECTIONS AND DRAINAGE TUBES.

By W. H. VITTUM, M.D., Baraboo, Wis.

Since the introduction of antiseptic surgery by Lister, the treatment of abscesses and suppurating sores of all descriptions has been far more satisfactory than formerly. The profession, with a few exceptions, at once saw and appreciated the advantages to be derived from a system of treatment which should exclude from the cavity of an abscess, or the surface of an ulcer, all those vegetable and animal sources of infection which are supposed to exercise so unfavorable an influence upon the healing process.

In a short time abcesses in nearly all parts of the body were treated by injections of carbolic acid. The pleural cavity was long an exception to this rule, and collections of pus in this situation were merely evacuated, or at most treated with slightly stimulant injections. Indeed, so recently has the antiseptic method of treatment been adopted, that most of our standard text books are utterly silent in regard to it. So recent a writer as Ashhurst says: "Boinet has recommended the injection of iodine into an empyemic cavity, and in one case in which I

saw this method employed, it certainly produced no undue irritation. I believe, however, that the advantages which were anticipated from this mode of treatment have been in most instances not fully realized." There is, however, neither here nor elsewhere in his book any mention in this connection of antiseptics strictly so called. Gross and Holmes, both in their old and new works, Bryant and Gant, of England, are all silent in regard to this mode of treatment.

Prof. F. H. Hamilton says, "abcesses resulting from the presence of foreign bodies in the cavity of the chest should occasionally be thoroughly syringed out with tepid water, or with such disinfectants as carbolic acid, chloride of sodium, or bromine." Why these most beneficial injections should be limited to those cases having a traumatic origin would seem incomprehensible.

In the Medical and Surgical History of the War of the Rebellion, page 581, I find the following commentary on the foregoing extract: "Surgeon F. H. Hamilton advised, in the cases complicated by the presence of foreign bodies in the cavity of the chest, thorough syringing 'with such disinfectants as carbolic acid, chloride of sodium, or bromine,' but no evidence of their efficacy is produced."

Of course no therapeutic measure can be rightly judged when it is applied only to those cases over whose complications it can, in the very nature of things, have no control.

In order to successfully disinfect the pleural cavity, drainage tubes of one form or another will have to be used. Prof. Gross throws the weight of his great learning and experience against the employment of this measure. He says: "The use of drainage tubes has lately been recommended under such circumstances, but the treatment, it seems to me, should not be encouraged, as it is both harsh and dangerous." These are, however, the sentiments of one of the opposers of antiseptic surgery in general, and as such need have no great weight with us; for if the beneficial effects of antiseptics are denied, there is no indication for either injections or drainage tubes.

Prof. E. Andrews, of Chicago, was probably the first among the surgeons of the west to propose injections of carbolic acid into an empyemic cavity. His first case occurred in June, 1872, and was reported in the *Chicago Medical Examiner* for December of that year, under the title, "Antiseptic Injections and Drainage Tubes in Empyema." Dr. Andrews says: "I conceived the idea because patients fared so badly by old methods, and because I found Lister's plan so successful in arresting suppuration in other parts of the body that I thought it might do equally well in the pleura."

Dr. Fraentzel, of Munich, first saw and used this method of treatment "in the beginning of the year 1873." I saw the operation performed at the Charity Hospital in New Orleans by the late Prof. Hawthorn, during the winter of 1874–5. I am told that it is now the regular treatment for empyema in New York city. I presume this is the case with the principal cities of the United States; of New Orleans and Chicago I can speak with certainty.

After this brief sketch of the history of antiseptic injections into the pleural cavity, let us glance for a moment at some of the details of the treatment. All of the authorities agree that the external opening should be a large one for the free exit of the pus. Fraentzel,' in his article on Pleuritis, in the 4th volume of Ziemssen's cyclopedia gives an elaborate account of what he terms the "radical operation" for empyema. His plan is to have a silver canula large enough to admit two Nélaton catheters at once, kept constantly in the wound. The cleansing and disinfecting the cavity is carried on by a force pump or syringe attached to one of these catheters and a suction pump to the other. The catheters are then passed together through the cannula and pressed deep down into the cavity of the chest, and the work of injection and withdrawal of the fluid accomplished by these means. The cannula is to be made of silver and has a collar which must accurately fit the outside of the patient's chest. Of course we living in the country, or even in large towns, can hardly have at our command the means necessary to accomplish this. But let us see whether a more simple contrivance will not answer the purpose. Prof. Andrews uses a simple rubber tube opening through a button of gutta percha. Prof. Hawthorn used a common flexible catheter for his injections, and maintained it *in situ* by means of adhesive strips. In a case which occurred in the practice of Dr. M. M. Davis and myself, we used a common flexible catheter brought through a circular disk of leather and sewed into the latter.

In regard to the fluid to be injected, there is some difference of opinion. Most surgeons think that a weak solution of carbolic acid, say two or three per cent., should be used at first, to be increased afterward if necessary. Dr. Fraentzel immediately after the operation uses a solution of common salt, and afterward some disinfectant. I quote from page 720, volume 4, of Ziemssen's cyclopedia: "After a few days, even if there is no fever, and the contents of the pleural sac are not fetid, we should replace the solution of common salt with compound tincture of iodine diluted with from twenty to fifty times its bulk of water, or we may use a solution of permanganate of potash, (one grain to the ounce,) or a solution of carbolic acid, (two grains to the ounce.)"

Prof. Hawthorn used a mixture of pure carbolic acid and water, (one part to six.) I saw him do this twice in patients enfeebled by the disease, and yet no injurious effects followed. I quote from a letter from this gentleman dated Dec. 6th, 1875: "I treated a child in private practice last spring with capital results. The child was three years old; had empyema; was aspirated; the fluid reaccumulated; a free incision was made, the chest washed out, a weak solution injected at first, but this failing, in the end a mixture of pure carbolic acid and water, (one part in six,) was used with effect of arresting suppuration. The mixture took the cuticle off the chest wall wherever it touched, but caused no ill symptoms within. The lung finally expanded and the child is now perfectly well." In the case already alluded to, occurring in the practice of Dr. Davis and myself, a ten per cent. mixture was used. The only unpleasant result was considerable dizziness, which, however, soon passed off, leaving no ill effects.

There is a difference of opinion as to whether any of the antiseptic fluid should be left in the cavity of the chest.

Fraentzel says the cavity should be completely emptied at each dressing. Prof. Hawthorn left some of the fluid from dressing to dressing. The latter plan was followed by Dr. Davis and myself. The pleura is probably so thickened by inflammation that no danger of absorption exists, and for a time, at least, the continuous action of the antiseptic can result in nothing but good.

It may not be amiss here to give a short account of the case which occurred to Dr. Davis and the writer, and which will show the general plan of treatment. Miss L. B., aged 22, on the 29th of Dec., 1869, had a severe attack of pleuritis which culminated in a purulent effusion. In April, 1870, the chest was punctured in the tenth intercostal space and injected with tincture of iodine, with no result except the establishment of a fistula. At the time when the patient came into our care she could not move without being thrown into a violent fit of coughing. The daily discharge of pus was from six to twelve ounces. She was much emaciated, and notwithstanding her excellent constitution, which had so long resisted this fearful drainage, was slowly sinking. The menses had been absent since the first attack of pluritis. Auscultation gave no sound over the left side of the chest, with the exception of a little space near the root of the lung, where bronchial breathing could be heard. The lung was evidently collapsed, and judging from the long continuance of effusion, most likely bound down by adhesions. The patient expectorated pus freely, showing a fistulous opening through the lung, which converted the case really into one of pyopneumothorax. I may remark in passing that this fistula closed in a few weeks from the date of operation. The patient had had a variety of treatment from different physicians, but notwithstanding this, had been unable to walk for three years and a half. We found a lateral curvature of the spine, resulting from her habitually sitting in a constrained position. There was also extreme deformity of the fingers and toes, the former being clubbed to a remarkable extent. On the 19th of Oct., 1875, we made a free incision into the plueral cavity in the site of the fistula and drew off twenty-four ounces of pus. We then introduced a drainage tube and washed out the cavity with tepid water. A mixture of carbolic acid and water, (one part in ten,) was then injected. The reduction in the amount of discharge was immediate and marked, hardly an ounce and a half of pus making its appearance during the twenty-four hours following the operation. This washing and injecting was repeated daily (varying the strength of the antiseptic mixture from time to time,) until Dec. 12th, when salicylic acid was substituted for the carbolic wash. During the whole time tonics and stimulants were freely administered. About Nov. 25th an attack of malarial fever supervened, which greatly augmented the amount of pus secreted. The fever, however, readily yielding to the usual remedies, the discharge at once subsided to its former quantity, and the patient has since then continued constantly to improve. On the 16th of November the menses appeared for the first time in nearly six years and have remained regular ever since. At the present time the discharge is virtually stopped. The cavity has a capacity of only four ounces and is steadily diminishing in size. Roughened and prolonged breathing can be heard over the upper part of the chest in front and over nearly the whole thoracic surface posteriorly, showing that the lung has, at least in part, re-expanded. The patient is strong, has a good appetite, and is able to take long walks in the open air.

In conclusion it may be well to give the results of such cases treated by antiseptic injections as I have been able to collect.

Prof. Andrews says, in a private letter: "I have tried it," (meaning this mode of treatment,) "in about six cases, and without a single failure, or a single bad symptom." Two cases that I saw treated at the Charity Hospital, in New Orleans, I understand to have recovered completely. These, with the case already quoted from Prof. Hawthorn's letter, make three cases for that gentleman. A case was related to me by Dr. C. Gapen which he treated while house surgeon of Cook County Hospital, in Chicago. The result was a perfect cure. Adding to these the case treated by Dr. Davis and the writer, and we have eleven cases with as many recoveries. Of course this remarkable success will not always follow the treatment, as will be shown by a statement of ten cases treated by Dr. Fraentzel. In ten cases he had five complete recoveries. The deaths in several instances, however, resulted from other causes than such as could be attributed in any way to the operation or after treatment. I shall take the liberty to quote at length his statement of these cases.

"Since the first case that I saw treated in this way in 1873, I have had the radical operation in eleven cases with purulent effusions, following the same principles both with regard to the operation and the after treatment. Complete recovery ensued in five. A sixth, in whose case the cannula had been removed somewhat too soon, and on account of whose foolish resistance, the strict after treatment had been interfered with, was dismissed with an incompletely healed thoracic fistula. He was afterwards for five months entirely without medical supervision, and was at last returned to the *Charité* with amyloid degeneration of the liver, spleen, and kidneys, and died there a few weeks ago. Four of those who had been operated upon died; one of ichorization of the pleural cavity with severe tubercular pleuritis and circumscribed caseous deposits in both lungs; another, who, at the time of the operation, was suffering from amyloid degeneration of the kidneys, and had a thoracic fistula, and for whom the radical operation with resection of a piece of rib, had been performed, died of secondary peritonitis; a third, who had been attacked with purulent pleuritis, as he was recovering from a severe form of typhoid, succumbed to an attack of pneumonia in the lower lobe of the uncompressed lung, which had probably arisen in consequence of catching cold during the operation; and finally the fourth patient, who in the course of a caseous pneumonia had become the victim of pyopneumothorax, and who had not been operated upon till some months later, when the purulent effusion began to increase considerably, sank from dysentery which set in the day after the operation, and which was at that time endemic in the hospital. An eleventh case was operated upon not quite a fortnight ago, and gives hope of recovery. This was a case in which an extensive purulent effusion supervened as an attack of pleuro-pneumonia, and after twice puncturing, re-accumulated in a very short time."

Dr. Fraentzel insists strenuously on his particular after-treatment, antiseptic injections and suitable drainage. These cases, ten of Dr. F.'s, and eleven before recited, give us twenty-one cases, with four deaths. At least two of these deaths cannot be ascribed to the operation, or even to the pre-existing empyema.

This record will speak for itself, especially when it is taken into consideration that the cases of the German professor occurred in a hospital where probably the opportunities for securing a good supply of fresh air, etc., were not nearly so great as they usually are in private practice.

The extreme simplicity of the operation and the utter harmlessness of judiciously employed anteseptic injections, will be strong reasons for the general adoption of this method of treating empyema.

